# Comparison of the Suitability Between NRS2002 and MUST as the First-Step Screening Tool for GLIM Criteria in Hospitalized Patients With GIST

**DOI:** 10.3389/fnut.2022.864024

**Published:** 2022-04-11

**Authors:** Xin Zhou, Junjin Liu, Qijuan Zhang, Siqi Rao, Xingye Wu, Jun Zhang, Juan Li

**Affiliations:** ^1^Department of Pharmacy, The First Affiliated Hospital of Chongqing Medical University, Chongqing, China; ^2^Department of Geriatrics, The First Affiliated Hospital of Chongqing Medical University, Chongqing, China; ^3^Department of Clinical Nutrition, The First Affiliated Hospital of Chongqing Medical University, Chongqing, China; ^4^Department of Gastrointestinal Surgery, The First Affiliated Hospital of Chongqing Medical University, Chongqing, China

**Keywords:** gastrointestinal stromal tumor (GIST), malnutrition, malnutrition risk, Global Leadership Initiative on Malnutrition (GLIM), Nutritional Risk Screening 2002 (NRS2002), Malnutrition Universal Screening Tool (MUST)

## Abstract

**Objective:**

The Global Leader Initiative on Malnutrition (GLIM) criteria have been recommended for malnutrition diagnosis recently, for which the first step is malnutrition risk screening with any validated tool. This study aims to investigate the incidence of malnutrition risk in gastrointestinal stromal tumor (GIST) inpatients and compare the suitability of Nutritional Risk Screening 2002 (NRS2002) and Malnutrition Universal Screening Tool (MUST) as the first-step screening tool for GLIM criteria.

**Methods:**

We retrospectively analyzed the clinical data of GIST inpatients in our hospital from January 2015 to December 2019. NRS2002 and MUST were used to screen malnutrition risk at the time of admission. The diagnostic consistency of these two tools with GLIM criteria for malnutrition was analyzed, and the predictive performance of both tools for the length of hospital stay and the occurrence of complications was also evaluated in surgical and non-surgical inpatients.

**Results:**

A total of 269 GIST inpatients were included in this study, of which 45.7 and 40.9% were at malnutrition risk determined by NRS2002 and MUST, respectively. In non-surgical inpatients, NRS2002 and MUST had similar diagnostic consistency with GLIM criteria in sensitivity (93.0 vs. 97.7%), specificity (81.1 vs. 81.1%), and Kappa value (*K* = 0.75 vs. 0.80), and high nutritional risk classified by NRS2002 and malnutrition identified by GLIM criteria were found to be associated with the length of hospital stay. In surgical inpatients, MUST had better diagnostic consistency with GLIM criteria in sensitivity (86.1 vs. 53.5%) and Kappa value (*K* = 0.61 vs. 0.30) than NRS2002, but no factors were found associated with the length of postoperative hospital stay or the occurrence of complications.

**Conclusion:**

The malnutrition risk is common in GIST inpatients. NRS2002 is more suitable than MUST for the first-step risk screening of the GLIM scheme in non-surgical inpatients, considering its better performance in screening malnutrition risk and predicting clinical outcomes. MUST was found to have good diagnostic consistency with GLIM criteria for malnutrition in both non-surgical and surgical GIST inpatients, and further studies need to be conducted to investigate its predictive performance on clinical outcomes.

## Introduction

Gastrointestinal stromal tumor (GIST) is the most common mesenchymal neoplasm in the digestive tract (1), which can rapidly turn from a potentially malignant tumor to cancer (2). As we know, patients with gastrointestinal tumors are usually accompanied by unbalanced nutritional status, such as protein deficiency and energy imbalance, and the incidence of malnutrition even reached from 40 to 80% (3). In general, malnutrition in patients with gastrointestinal tumors was correlated with shorter survival time, poorer tolerance to chemotherapy, and worse quality of life (4–6). If malnutrition, especially undernutrition, has not been identified and corrected in time, it could lead to reduced immune function, increased infectious complications, prolonged hospitalization, increased mortality rates of patients, and more associated medical expenditure (7–9). Therefore, it is of great significance to make an accurate assessment of the nutritional status in time, and then draw up an individualized plan for the nutritional treatment, thereby improving or even reversing the clinical outcomes of patients with gastrointestinal tumors (10).

Although the diagnosis of malnutrition is the basis of clinical nutrition intervention, there were no recognized definitions and uniform diagnostic criteria for malnutrition for quite a long time around the world. Recently, the global (nutrition) leaders launched the criteria for the diagnosis of malnutrition, referred to as Global Leader Initiative on Malnutrition (GLIM) criteria. According to GLIM criteria, malnutrition can be diagnosed by conforming to at least one phenotypic criterion (involuntary weight loss, low body mass index, and muscle loss) and one etiologic criterion (reduced food intake or assimilation and inflammation or disease burden) (11).

A consensus scheme for malnutrition diagnosis in adults was also proposed by GLIM, of which the first step is malnutrition risk screening with any validated tool, and the second step is assessment for diagnosis and severity grading of malnutrition (11). Over the past decades, several screening tools have been introduced and evaluated. Among them, two tools commonly used in clinical practice are Nutritional Risk Screening 2002 (NRS2002) and Malnutrition Universal Screening Tool (MUST) (12, 13). NRS2002 is the tool proposed by the European Society for Clinical Nutrition and Metabolism (ESPEN) and mainly applied to inpatients for screening the indications for nutritional support (14). MUST was developed by the British Association for Parenteral and Enteral Nutrition (BAPEN) to detect the malnutrition risk for all adult patients in different medical institutions (15). Compared with MUST, the NRS2002 scoring system contains the nutritional components of MUST, and in addition, grading of severity of disease as a reflection of increased nutritional requirements, and the age assessment (14).

At present, the management of GIST inpatients has been mainly focused on surgery and medication, and the nutritional status of GIST inpatients has been less explored. Therefore, this study aims to investigate the incidence of malnutrition risk by the use of NRS2002 and MUST, as well as to compare the suitability of these two tools as the first-step screening tool for GLIM criteria in GIST inpatients.

## Methods and Materials

### Study Design and Participants

This study retrospectively analyzed the clinical data of GIST inpatients in our hospital from January 2015 to December 2019. Participants were patients with GIST admitted to the department of gastrointestinal surgery in the First Affiliated Hospital of Chongqing Medical University during that period. This study was performed in accordance with the World Medical Association Declaration of Helsinki and approved by the Medical Ethical Committee of the First Affiliated Hospital of Chongqing Medical University. All the patients included in this study provided informed consent.

The inclusion criteria were as follows: (1) pathologically confirmed GIST; (2) aged 18–90 years; (3) hospitalized for more than 24 h; (4) no emergency surgery within 24 h after admission; and (5) willing to accept malnutrition risk screening and assessment. Patients were excluded if it was difficult to obtain their accurate height and weight, perform body composition measurements on them, or they could not complete malnutrition risk screening and assessment.

### Data Collection

The collected data mainly included the following aspects: patient general information, anthropometric measurement, implementation of malnutrition screening and malnutrition assessment.

Patient general information were obtained from medical records, which included age, sex, medical treatment history, laboratory examination and combined symptoms at admission, primary tumor site, tumor risk stratification, length of hospital stay, or complications, during hospitalization.

Anthropometric measurements included body weight, height, body mass index (BMI), and muscle mass, which were carried out by a trained nurse or dietician. The weight and height of the patients were measured by using uniform calibrated instruments on the morning within 24 h after admission. After overnight fasting, emptying of bowels, and urinating in the morning, the patients wearing hospital gowns were weighed before breakfast without shoes. Meanwhile, barefoot heights were measured. BMI was calculated using the standard formula as weight (kg) divided by the square of height (m^2^). Muscle mass was determined by fat free mass (FFM), which was obtained through bioelectrical impedance measured by InBodyS10. Fat free mass index (FFMI) was calculated as FFM divided by the square of height (m^2^).

Malnutrition risk of GIST inpatients was screened with NRS2002 and MUST, which was performed by a trained nutritional support pharmacist within 48 h of admission. The NRS2002 scoring system consists of three parts according to ESPEN guidelines (14). The first part of NRS2002 assesses the nutritional status of the patient, which is based on the changes in weight in the recent 3 months, dietary intake one-week before hospitalization, and the BMI. The second part of the NRS2002 assesses the severity of the disease, which could be scored by its impact on the increased nutritional requirements of patients. The scores for the first two parts of the NRS2002 vary from 0 to 3. The last part of the NRS2002 is the age assessment. If the patient is 70 years or older, add 1 score. Therefore, the final score of NRS2002 can range from 0 to 7. Patients with a total NRS2002 score of ≥ 3 indicate a high nutritional risk. The MUST was also used to screen malnutrition risk in this study, which includes three components, such as BMI (in kg/m^2^), unplanned weight loss in past 3–6 months, and absence or inadequacy of dietary intake for > 5 d due to the presence of acute disease (15). The score for each component varies from 0 to 2. Overall risk of malnutrition according to MUST is rated as low (score = 0), medium (score = 1), or high (score ≥ 2).

**Figure 1:**
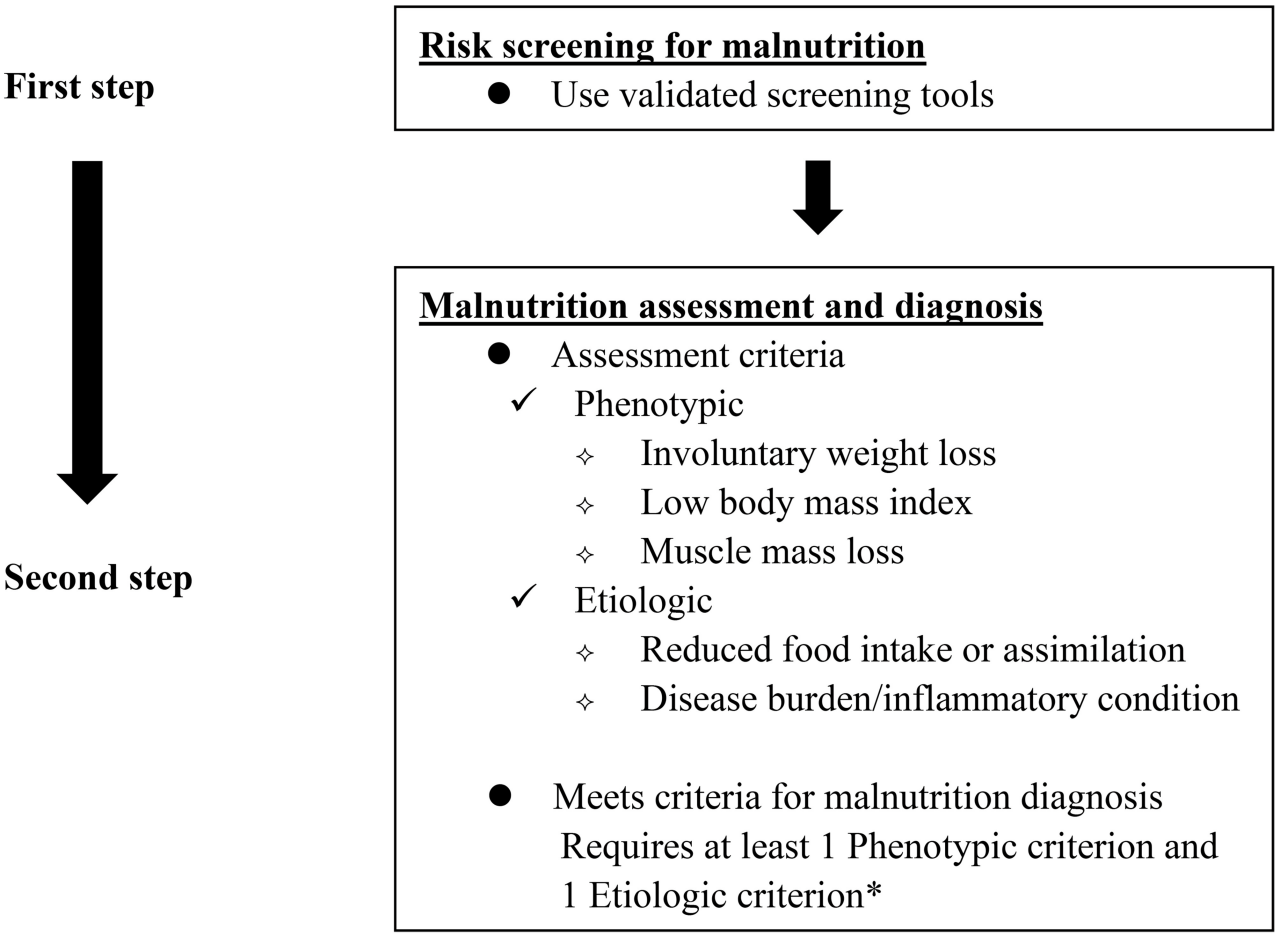
GLIM diagnostic scheme for screening, assessment, and diagnosis of malnutrition. *In this study, patients diagnosed with GIST were all thought to meet the etiologic criterion as GLIM consensus mentioned that most chronic organ diseases, such as cancer, are associated with chronic or recurrent inflammation of a mild to moderate degree.

According to GLIM criteria (11), malnutrition can be diagnosed for patients by conforming to at least one phenotypic criterion and one etiologic criterion based on the first step of malnutrition risk screening (Figure 1). To assess the diagnostic consistency between the two risk-screening tools and GLIM criteria for malnutrition in this study, malnutrition was directly diagnosed according to GLIM criteria without the first step of malnutrition risk screening. Phenotype criteria include: (1) involuntary weight loss: > 5% within 6 months or > 10% beyond 6 months; (2) low BMI for Asian: < 18.5 kg/m^2^ if < 70 years or < 20 kg/m^2^ if > 70 years; and (3) reduced muscle mass: a low FFMI (< 15 kg/m^2^ for women, < 17 kg/m^2^ for men). Etiological criteria include: (1) < 50% of energy requirements > 1 week, or any reduction for at least 2 weeks, or any chronic gastrointestinal symptoms that lead to inadequate or impaired absorption and assimilation in patients; (2) inflammation associated with acute disease/injury or chronic disease. As GLIM consensus mentioned that most chronic organ diseases, such as cancer, are associated with chronic or recurrent inflammation of a mild to a moderate degree, patients diagnosed with GIST in this study were all thought to meet the etiologic criterion.

### Statistical Analysis

Statistical evaluations of the two risk-screening tools (i.e., MUST and NRS2002) compared with the GLIM criteria for malnutrition diagnosis were performed in both total sample and subgroups (with surgery or not suitable for surgery because of metastatic or advanced GIST). Continuous variables were expressed as mean values ± SD (mean ± SD), while categorical variables were presented as absolute values and percentages. Differences between variables were tested with the use of the Mann–Whitney *U*-test or the chi-square test depending on data characteristics. Multivariable logistic regressions were used to identify the factors associated with the length of hospital stay or the occurrence of complications.

Cohen's Kappa statistic was calculated to determine the diagnostic consistency between the screening tools (i.e., MUST and NRS2002) and GLIM criteria for the identification of malnutrition according to some studies (12, 16, 17). The kappa value can be interpreted as follows: 0–0.20 as no agreement, 0.21–0.39 as minimal, 0.40–0.59 as weak, 0.60–0.79 as moderate, 0.80–0.90 as strong, and above 0.9 as almost perfect agreement (18). The sensitivity, specificity, positive predictive value (PPV), negative predictive value (NPV), positive (LR+), and negative (LR-) likelihood ratio of the two tools were also evaluated referred to the GLIM criteria. Meanwhile, the receiver operating characteristic (ROC) curve of the two tools was also used to determine the ability to accurately distinguish patients with malnutrition from well nutrition according to some studies (12, 16, 17). Accuracy is considered low when the area under the ROC curve varies from 0.50 to 0.69, moderate from 0.70 to 0.90, and high if more than 0.9 (17).

Statistical analysis was performed by SPSS for Windows (version 22.0, IBM Corp., Armonk, NY, USA), and *P* < 0.05 were considered statistically significant (two-tailed).

## Results

### Patient Characteristics

At the time of data analysis, a total of 301 GIST inpatients from January 2015 to December 2019 were alive, of which 7 inpatients were excluded because of emergency surgery on the day of admission and 25 inpatients were excluded because they refused to accept risk screening and assessment for malnutrition. Finally, 269 inpatients were included. The median age was 57 years (range: 29–90), with a sex ratio of 1.07:1 (51.7% were women). The characteristics of the total, surgical, and non-surgical participants were shown in Table 1.

**Table 1:** Characteristics of participants.

	**Total patients**			**Surgical patients**			**Non-surgical patient**		
	**Well-nourished (*n* = 183)**	**Malnourished (*n* = 86)**	***P* value**	**Well-nourished (*n* = 146)**	**Malnourished (*n* = 43)**	***P* value**	**Well-nourished (*n* = 37)**	**Malnourished (*n* = 43)**	***P* value**
**Age, y**	55.93 ± 11.34	59.23 ± 13.65	0.054	55.64 ± 38.18	58.42 ± 43.13	0.297	57.11 ± 10.20	60.05 ± 10.76	0.214
**Sex**, ***n*** **(%)**									
Male	90 (69.2%)	40 (30.8%)	0.683	68 (79.1%)	18 (20.9%)	0.585	22 (50.0%)	22 (50.0%)	0.457
Female	93 (66.9%)	46 (33.1%)		78 (75.7%)	25 (24.3%)		15 (41.7%)	21 (58.3%)	
**BMI (kg/m**^2^)	23.73 ± 2.40	19.98 ± 2.62	<0.001	23.86 ± 8.23	20.33 ± 5.04	<0.001	23.23 ± 2.09	19.64 ± 3.22	<0.001
**Albumin (g/L)**	39.46 ± 6.18	35.77 ± 7.25	<0.001	39.43 ± 6.34	37.91 ± 7.44	0.227	39.59 ± 5.55	33.63 ± 6.45	<0.001
**Hemoglobin (g/L)**									
Male	116.61 ± 31.47	100.8 ± 30.95	<0.01	118.97 ± 31.17	106.39 ± 28.75	0.116	109.32 ± 31.99	96.27 ± 32.57	0.187
Female	108.00 ± 23.75	99.90 ± 28.00	0.098	109.62 ± 23.41	98.76 ± 30.31	0.11	101.29± 25.57	99.40 ± 24.44	0.825
**Primary tumor site**, ***n*** **(%)**								
Stomach	91 (68.4%)	42 (31.6%)	0.943	78 (76.5%)	24 (23.5%)	0.742	15 (45.5%)	18 (54.5%)	0.196
Small intestine	60 (66.7%)	30 (33.3%)		52 (78.8%)	14 (21.2%)		8 (33.3%)	16 (66.7%)	
Colorectum	14 (73.7%)	5 (26.3%)		10 (71.4%)	4 (28.6%)		4 (80.0%)	1 (20.0%)	
Others	18 (66.7%)	9 (33.3%)		8 (88.9%)	1 (11.1%)		10 (55.6%)	8 (44.4%)	
**Tumor risk stratification**, ***n*** **(%)**								
High risk	89 (61.8%)	55 (38.2%)	0.112	69 (75.8%)	22 (24.2%)	0.817	20 (37.7%)	33 (62.3%)	0.121
Medium risk	34 (73.9%)	12 (26.1%)		29 (80.6%)	7 (19.4%)		5 (50.0%)	5 (50.0%)	
Low risk	43 (78.2%)	12 (21.8%)		39 (79.6%)	10 (20.4%)		4 (66.7%)	2 (33.3%)	
Very low risk	17 (70.8%)	7 (29.2%)		9 (69.2%)	4 (30.8%)		8 (72.7%)	3 (27.3%)	
**Targeted Therapy**, ***n*** **(%)**									
Yes	26 (44.1%)	33 (55.9%)	<0.001	12 (92.3%)	1 (7.7%)	0.318	14 (30.4%)	32 (69.6%)	0.001
No	157 (74.8%)	53 (25.2%)		134 (76.1%)	42 (23.9%)		23 (67.7%)	11 (32.4%)	
**Number of combined symptoms**, ***n*** **(%)**							
0	41 (87.2%)	6 (12.8%)	<0.001	34 (87.2%)	5 (12.8%)	0.064	7 (87.5%)	1 (12.5%)	0.023
1	96 (70.6%)	40 (29.4%)		75 (79.8%)	19 (20.2%)		21 (50.0%)	21 (50.0%)	
2	39 (54.2%)	33 (45.8%)		31 (68.9%)	14 (31.1%)		8 (29.6%)	19 (70.4%)	
3	7 (50.0%)	7 (50.0%)		6 (54.6%)	5 (45.5%)		1 (33.3%)	2 (66.7%)	
**Length of stay in hospital, days**							6.05 ± 2.50	12.21 ± 3.75	<0.001
**Postoperative stay in hospital, days**				8.24 ± 4.24	9.65 ± 11.31	0.119			
**Complications**, *n* (%)									
Yes				20 (71.4%)	8 (28.6%)	0.426	2 (33.3%)	4 (66.7%)	0.815
No				126 (78.3%)	35 (21.7%)		35 (47.3%)	39 (52.7%)	

*Malnutrition is identified according to Global Leader Initiative on Malnutrition (GLIM) criteria. BMI, body mass index. Continuous variables were expressed as mean values ± standard deviation (mean ± SD), and categorical variables were presented as absolute values and percentages (n, %). P values were determined by the use of the Mann–Whitney U-test or the chi-square test, and P values < 0.05 were considered statistically significant*.

In this study, we found that malnourished patients had a significantly lower BMI in total, non-surgical, and surgical GIST inpatients (*P* < 0.001), a significantly lower level of serum albumin in total and non-surgical GIST inpatients (*P* < 0.001), and a significantly longer length of hospital stay in non-surgical GIST inpatients (*P* < 0.001) as compared with well-nourished patients. In addition, GIST patients with targeted therapy had a higher incidence of malnutrition compared to the patients with no targeted therapy (*P* < 0.001 for total patients and *P* = 0.001 for non-surgical patients). With the number of combined symptoms at admission increasing, the incidence of malnutrition increased significantly (*P* < 0.001 for total patients and *P* = 0.023 for non-surgical patients).

### Consistency Analysis Between Malnutrition Screening Tools and GLIM Criteria

Table 2 showed the details of the classifications for malnutrition risk determined by NRS2002 and MUST. The incidence of moderate/high risk of malnutrition determined by NRS2002 and MUST was 45.7 and 40.9% for total inpatients, 58.8 and 61.3% for non-surgical inpatients, and 40.2 and 32.3% for surgical in patients.

**Table 2:** Cross-tabulation results of the malnutrition identified by GLIM criteria and the malnutrition risk classified by NRS2002 and MUST.

		**Total patients**		**Surgical patients**		**Non-surgical patients**	
		**GLIM criteria**		**GLIM criteria**		**GLIM criteria**	
		**Malnourished**	**Well-nourished**	**Malnourished**	**Well-nourished**	**Malnourished**	**Well-nourished**
NRS2002	high nutritional risk	70	53	30	46	40	7
	Low nutritional risk	16	130	13	100	3	30
MUST	moderate/high nutritional risk	79	31	37	24	42	7
	Low nutritional risk	7	152	6	122	1	30

*GLIM, Global Leader Initiative on Malnutrition; MUST, Malnutrition Universal Screening Tool; NRS2002, Nutritional Risk Screening 2002*.

*Score of NRS2002 ≥ 3 is considered to be at a high nutritional risk. Score of MUST ≥ 1 is considered to be at a moderate/high nutritional risk*.

Table 3 showed the evaluation of diagnostic consistency for malnutrition between the two screening tools and the GLIM criteria. In total inpatients, MUST had a higher value of sensitivity (91.9 vs. 65.1%), PPV (71.8 vs. 56.9%), NPV (95.6 vs. 89.0%), Kappa (*K* = 0.70 vs. 0.47), and a larger area under the ROC curve (AUC = 0.90 vs. 0.83) compared with NRS2002.

**Table 3:** Statistical evaluations of the two risk-screening tools compared with GLIM criteria for the diagnosis of malnutrition.

	**Total patients (*****n*** **=** **269)**		**Surgical patients (*****n*** **=** **189)**		**Non-surgical patients (*****n*** **=** **80)**	
	**NRS2002**	**MUST**	**NRS2002**	**MUST**	**NRS2002**	**MUST**
Sensitivity (%)	65.1	91.9	53.5	86.1	93.0	97.7
Specificity (%)	90.7	83.1	89.0	83.6	81.1	81.1
Positive predictive value (%)	56.9	71.8	39.5	60.7	85.1	85.7
Negative predictive value (%)	89.0	95.6	88.5	95.3	90.9	96.8
Positive likelihood ratio	7.01	5.42	4.88	5.23	4.92	5.16
Negative likelihood ratio	0.39	0.10	0.52	0.17	0.09	0.03
Kappa value (95%CI)	0.47 (0.37–0.57)^*^	0.70 (0.61–0.79) ^*^	0.30 (0.17–0.43) ^*^	0.61 (0.48–0.73) ^*^	0.75 (0.60–0.89) ^*^	0.80 (0.67–0.93) ^*^
AUC	0.83	0.90	0.75	0.86	0.94	0.94

*GLIM, Global Leader Initiative on Malnutrition; MUST, Malnutrition Universal Screening Tool; NRS2002, Nutritional Risk Screening 2002; CI, Confidence interval; AUC, Area under the receiver operating characteristic curve*;

* *P < 0.001*.

**Figure 2:**
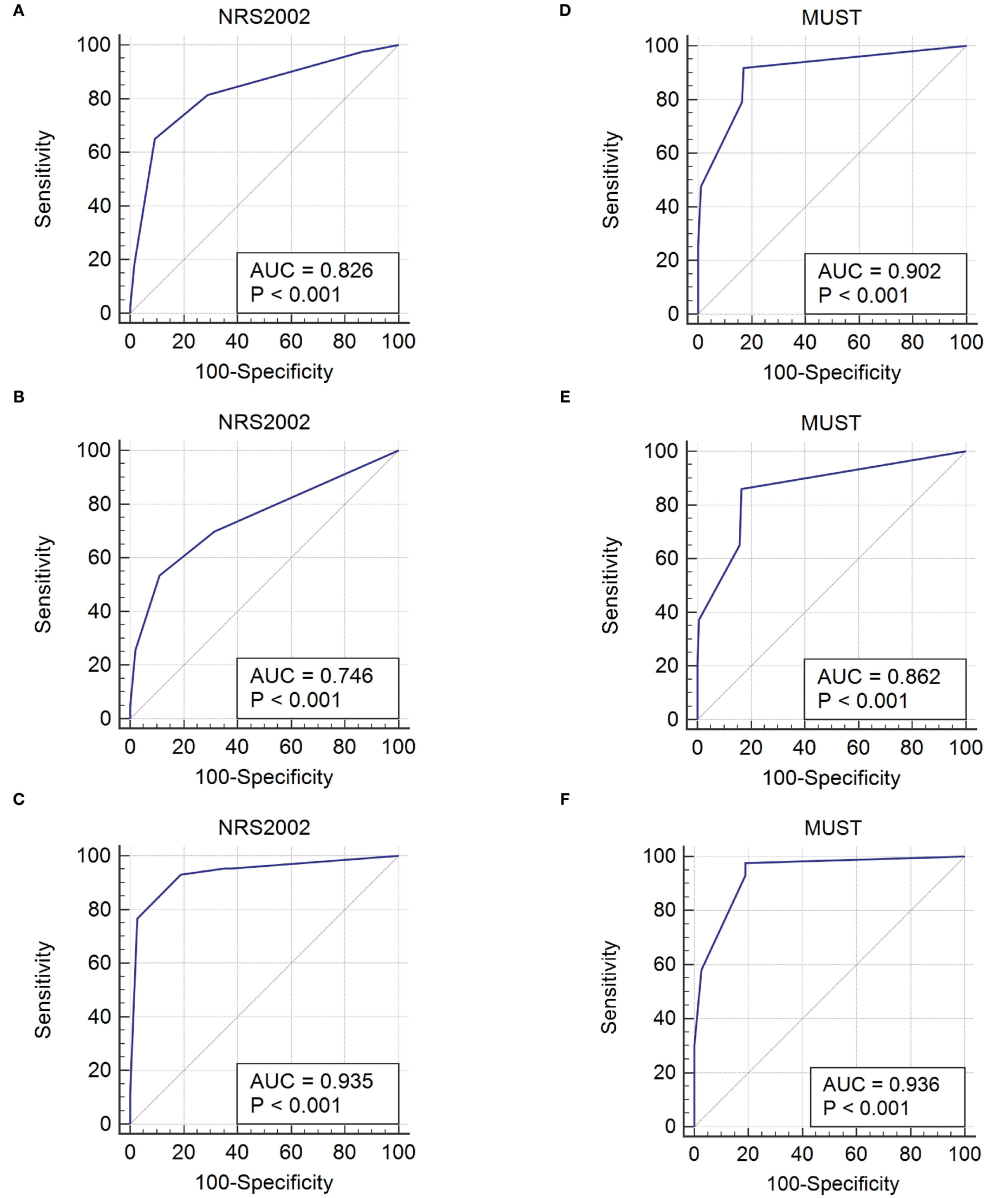
Diagnostic accuracy of NRS2002 compared to GLIM criteria for malnutrition in total **(A)**, surgical **(B)**, and non-surgical **(C)** GIST inpatients. Diagnostic accuracy of MUST compared to GLIM criteria for malnutrition in total **(D)**, surgical **(E)**, and non-surgical **(F)** GIST inpatients. NRS2002, Nutritional Risk Screening 2002; MUST, Malnutrition Universal Screening Tool; AUC, Area under the receiver operating characteristic curve; GLIM, Global Leadership Initiative on Malnutrition.

Considering that there might be some heterogeneity between non-surgical and surgical inpatients, we performed subgroup analyses to further evaluate the accuracy of NRS2002 and MUST in light of GLIM criteria for the malnutrition diagnosis (Table 3). In the non-surgical inpatients, NRS2002 and MUST had similar consistency with GLIM criteria in sensitivity (93.0 vs. 97.7%), specificity (81.1 vs. 81.1), PPV (85.1 vs. 85.7%), NPV (90.9 vs. 96.8%), Kappa value (*K* = 0.75 vs. 0.80), and area under the ROC curve (AUC = 0.94 vs. 0.94). In surgical inpatients, MUST had a higher value of sensitivity (86.1 vs. 53.5%), PPV (60.7 vs. 39.5%), NPV (95.3 vs. 88.5%), Kappa (*K* = 0.61 vs. 0.30), and a larger area under the ROC curve (AUC = 0.86 vs. 0.75) compared with NRS2002. ROC curves were presented in Figure 2.

### Univariate and Multivariate Analyses for Factors Associated With the Length of Hospital Stay or the Occurrence of Complications

In the univariate analysis of complications occurrence, gender was the only associated factor in surgical inpatients (OR = 2.96, *P* = 0.013), and no statistical difference was found in other factors among surgical or non-surgical inpatients.

Table 4 showed the univariate and multivariate analyses for the length of hospital stay in non-surgical inpatients and postoperative hospital stay in surgical inpatients. In non-surgical inpatients, the associated factors in the univariate analysis for the length of hospital stay were BMI, serum albumin, targeted therapy, number of combined symptoms at admission, malnutrition risk classified by NRS2002 or MUST, malnutrition identified by GLIM criteria, of which high risk of malnutrition classified by NRS2002 and malnutrition identified by GLIM criteria were found to be associated with the length of hospital stay ≥ 9 d (median hospital stay = 9 d) in the multivariate analysis. In the univariate analysis for the length of postoperative hospital stay in surgical inpatients, the associated factors were serum albumin, hemoglobin, primary tumor site, tumor risk stratification, and malnutrition risk classified by NRS2002. However, no significant associated factor was found in the multivariate analysis.

**Table 4:** Univariate and multivariate analyses for the length of hospital/postoperative hospital stay in non-surgical/surgical patients with GIST.

**Variable**	**Surgical patients (*****n*** **= 189, median postoperative hospital stay = 7)**				**Non-surgical patients (*****n*** **= 80, median hospital stay = 9)**			
	**Univariate**		**Multivariate**		**Univariate**		**Multivariate**	
	**OR (95%CI)**	***P* value**	**OR (95%CI)**	***P* value**	**OR (95%CI)**	***P* value**	**OR (95%CI)**	***P* value**
**Age**	0.99 (0.97–1.02)	0.623			1.02 (0.98–1.07)	0.348		
**Sex**	1.16 (0.65–2.05)	0.623			0.43 (0.17–1.06)	0.067		
**BMI**	0.98 (0.89–1.09)	0.742			0.77 (0.65–0.91)	0.002	1.31 (0.89–1.94)	0.173
**Albumin**	0.92 (0.87–0.97)	<0.001	0.94 (0.88–1.00)	0.054	0.86 (0.79–0.94)	<0.001	0.96 (0.84–1.10)	0.529
**Hemoglobin**	0.99 (0.98–1.00)	0.027	1.00 (0.99–1.02)	0.821	0.99 (0.98–1.01)	0.273		
**Primary tumor site**		0.024		0.457		0.358		
Small intestine vs. Stomach	2.57 (1.35–4.86)	0.004	0.64 (0.79–3.42)	0.187	1.39 (0.48–4.06)	0.549		
colorectum vs. Stomach	1.56 (0.51-4.80)	0.435	1.61 (0.48-5.40)	0.444	0.21 (0.02-2.07)	0.181		
Others vs. Stomach	3.13 (0.74-13.2)	0.121	2.45 (0.52-11.7)	0.26	0.67 (0.21-2.12)	0.491		
**Tumor risk stratification**		0.001		0.106		0.183		
Very low risk vs. High risk	0.16 (0.04–0.63)	0.009	0.27 (0.06–1.12)	0.071	0.38 (0.10–1.44)	0.153		
Low risk vs. High risk	0.29 (0.14-0.60)	0.001	0.45 (0.20-1.00)	0.051	0.13 (0.01-1.20)	0.073		
Medium risk vs. High risk	0.35 (0.16–0.77)	0.009	0.50 (0.20–1.22)	0.128	0.66 (0.17–2.55)	0.534		
**Targeted Therapy**	0.27 (0.07–1.01)	0.051		0.103	0.36 (0.15–0.91)	0.030	1.39 (0.22–8.93)	0.728
**Number of combined symptoms**	1.35 (0.94–1.94)	1			2.11 (1.06–4.20)	0.033	0.85 (0.24–3.03)	0.798
**NRS2002 (**≥ 3)	0.51 (0.28–0.92)	0.025	0.71 (0.37–1.37)	0.307	0.03 (0.01–0.12)	<0.001	0.05 (0.00–0.81)	0.036
**MUST (**≥ 1)	0.68 (0.37–1.25)	0.216			0.04 (0.01–0.15)	<0.001	9.87 (0.29–339)	0.205
**GLIM**	0.80 (0.41–1.58)	0.523			0.02 (0.00–0.06)	<0.001	0.01 (0.00–0.11)	0.001

*Variables were analyzed between greater or less than the median postoperative hospital stay/hospital stay. Variables showed statistically significance in univariate analysis were entered into a multivariate analysis with binary logistic regression. GLIM, Global Leader Initiative on Malnutrition; MUST, Malnutrition Universal Screening Tool; NRS2002, Nutritional Risk Screening 2002; OR, odds ratio; CI, Confidence interval. P < 0.05 were considered statistically significant*.

## Discussion

Recently, the GLIM criteria have been recommended for the malnutrition diagnosis, and a two-step model for risk screening and diagnostic assessment was also proposed by GLIM consensus. In this study, we investigated the incidence of malnutrition risk in GIST inpatients, and compared the suitability of NRS2002 and MUST as the first-step screening tool for GLIM criteria in GIST inpatients in view of their screening and predictive performance.

### Incidence of Malnutrition Risk in GIST Inpatients

GIST is the most common mesenchymal neoplasm in the digestive tract (1). However, few people paid attention to the nutritional status of GIST patients and related studies were limited. Therefore, we investigated the incidences of malnutrition risk in GIST inpatients. As we know, malnutrition risk screening was performed only with NRS2002 in GIST patients in previous studies (19, 20). The incidence of malnutrition risk for total GIST inpatients in our study (46% with NRS2002 and 41% with MUST, respectively) was lower than 78% reported by Ding et al. (19), but higher than 34% reported by Yin et al. (20). In the study of Ding et al. (19), the average age of patients was higher than that in our study and more patients had a BMI <18.5 kg/m^2^, which were both important components of the NRS2002 scoring system. That might explain the difference in the incidence of malnutrition risk between the two studies. In the study of Yin et al. (20), the incidence of malnutrition risk was lower in patients with intermediate-risk of tumor stratification than that in our study. The reason for this difference might be that most patients with intermediate-risk admitted to our hospital were in serious condition, while the patients with mild illness were not admitted to the hospital for having been treated in the daily follow-up.

### Comparison of Screening and Predictive Performance Between NRS2002 and MUST

A consensus scheme consisting of risk screening and assessment for the malnutrition diagnosis was proposed by GLIM, and there was a strong consensus that the key first step in the evaluation of nutritional status is malnutrition risk screening to identify “at-risk” status by the use of any validated screening tool (11). As observed in this study, there was a difference between the incidences of malnutrition risk identified by NRS2002 and MUST for total, non-surgical, or surgical GIST inpatients. Considering that there might be some heterogeneity between non-surgical and surgical inpatients, the suitability of NRS2002 and MUST as the first-step screening tool for GLIM criteria in GIST was further evaluated in these two types of patients, respectively. Given the screening tool with better diagnostic consistency with GLIM criteria might indicate its better application in the GLIM scheme, we firstly compared the screening performance of NRS2002 and MUST on malnutrition in light of the GLIM criteria, which could be quantitated by measures, such as sensitivity, specificity, PPV, NPV, LR+, LR-, and area under the ROC curve (21). Besides that, we also evaluated the predictive performance of NRS2002 and MUST on the length of hospital stay and the occurrence of complications, for better prediction of meaningful health outcomes that are known to be associated with malnutrition (22).

For non-surgical inpatients included in this study, NRS2002 and MUST had similar consistency with GLIM criteria in sensitivity, specificity, PPV, NPV, Kappa value, and area under the ROC curve. However, only malnutrition risk classified by NRS2002 and malnutrition diagnosed by GLIM criteria were found to be associated with the length of hospital stay in the multivariate analysis. This result can be attributed to differences in the original design of the NRS2002 and MUST. The purpose of the NRS2002 is to screen patients who might benefit from receiving nutritional support (14), while MUST is developed for the screening of malnutrition (14, 15). In some studies (23, 24), NRS2002 had been suggested as a strong predictor of clinical outcomes, such as length of hospital stays, complications, and mortality. Thus, NRS2002 might be suitable as the first-step screening tool for GLIM criteria in non-surgical GIST inpatients considering its screening and predictive performance. In addition, it was also found that the OR values for MUST in non-surgical GIST patients differed greatly in the univariate and multivariate analyses for the length of hospital stay. This might be because of the influence of other covariates in multivariate analysis and the uneven distribution of the raw data. Therefore, further prospective studies are needed to confirm whether moderate/high nutritional risk classified by MUST is associated with the length of hospital stay or other indicators for clinical outcomes.

For surgical GIST inpatients included in this study, MUST showed better consistency with GLIM criteria manifested as a higher value of sensitivity, PPV, NPV, Kappa value, and a larger area under the ROC curve. As we mentioned in the method, patients diagnosed with GIST in this study were all thought to meet the etiologic criterion. Thus, the consistency between NRS2002/MUST and GLIM criteria mainly depends on the comparison between the components of the two screening tools and the phenotypic indicators of GLIM criteria. Since NRS2002 is designed to screen patients who may benefit from nutritional support, the disease score of NRS2002 increased by 2 points if the patient planned to undergo major abdominal surgery. As a result, the patient would be classified as high malnutrition risk even without weight loss, low BMI, or reduced muscle mass, if he or she was ≥ 70 years old, which leads to poor consistency with GLIM criteria. A similar result was observed by Xu et al. in surgical patients that more than half of patients with malnutrition risk screened by NRS2002 did not meet the GLIM criteria for malnutrition (25). In the previous studies, MUST was found to have a high sensitivity and specificity and excellent diagnostic accuracy for the identification of malnutrition in patients with geriatric gastrointestinal cancer and patients with colorectal cancer (13, 26). In this study, MUST was also found to have good consistency with GLIM criteria for malnutrition diagnosis in surgical GIST inpatients. However, malnutrition risk classified by MUST did not show a correlation with the length of postoperative hospital stay or the occurrence of complications in this study. For these reasons, the predictive value of MUST for clinical outcomes in surgical GIST inpatients needs to be further verified in combination with other meaningful health outcomes associated with malnutrition.

### Strengths and Limitations

The strength of our study is that hospitalized patients with GIST were divided into surgical and non-surgical inpatients to compare the suitability of NRS2002 and MUST as the first-step screening tool for GLIM criteria in view of the screening and predictive performance of the two risk-screening tools. In our study, different results were obtained for surgical and non-surgical inpatients, and it may provide a better reference for clinical practice by considering both the screening and predictive performance of these two tools.

As the limitation of this study, the etiologic components of the GLIM criteria were determined only based on the cancer diagnosis without a clarification of disease severity and the reduction of food intake or assimilation, which may have some impacts on the results. Thus, we plan to carry out a prospective study to find a more accurate diagnostic combination in the future since different combinations of phenotypic and etiologic criteria can identify malnutrition according to GLIM criteria. In addition, we did not investigate the relationships between the malnutrition risk classified by NRS2002 or MUST and other indicators for clinical outcomes, such as survival time and mortality, in patients with GIST, which would be implemented after we collect enough data.

## Conclusion

The malnutrition risk is common in GIST inpatients. NRS2002 is more suitable than MUST for the first-step risk screening of the GLIM scheme in non-surgical inpatients, considering its better performance in screening malnutrition risk and predicting clinical outcomes. MUST was found to have good diagnostic consistency with GLIM criteria for malnutrition in both non-surgical and surgical GIST inpatients, and further studies need to be carried out to investigate its predictive performance on clinical outcomes.

## Data Availability Statement

The raw data supporting the conclusions of this article will be made available by the authors, without undue reservation.

## Ethics Statement

The studies involving human participants were reviewed and approved by the Medical Ethical Committee of the First Affiliated Hospital of Chongqing Medical University. The patients/participants provided their written informed consent to participate in this study.

## Author Contributions

JLi contributed to conception, design, administration, validation, writing, and editing of the manuscript. XZ contributed to writing, malnutrition risk screening, and assessment. JLiu and XW contributed to data collection and analysis. QZ contributed to body composition measurement. SR contributed to weight and height measurements. JZ contributed to the provision of patients. All authors contributed to the article and approved the submitted version.

## Funding

This work was supported by the Chongqing Municipal Health Commission (grant number: 2021MSXM335).

## Conflict of Interest

The authors declare that the research was conducted in the absence of any commercial or financial relationships that could be construed as a potential conflict of interest.

## Publisher's Note

All claims expressed in this article are solely those of the authors and do not necessarily represent those of their affiliated organizations, or those of the publisher, the editors and the reviewers. Any product that may be evaluated in this article, or claim that may be made by its manufacturer, is not guaranteed or endorsed by the publisher.
